# Paralysis Following Dentition

**Published:** 1871-06

**Authors:** 


### ARTICLE XI.

### *Paralysis Following Dentition.*

George Hoover, æt. two years and four months, is a child very small, very short, and lacking power in his lower extremities. This child, according to the statement of the father, weighed eight pounds at birth, and seven and a half pounds nine months after ; not much of a gain !

The child suffered from spasms when a year old. Its teeth, dentition being in progress at present, are in bad condition. Otherwise than I have mentioned the child seems well. His intellect good. How then can we account for its present helplessness? While we sum up the case we glean the following facts. This attack came on suddenly. The child was teething and during the day had seemed perfectly healthy. It awoke early the following morning with coated tongue, excessive thirst dry skin, and all the other attendants of high fever, *and was found to be unable to maintain itself upon its feet.* As the case unfavorably progressed the muscles became soft and atrophied, and ultimately underwent partial fatty degeneration. The legs became flabby and cold, but while the lower extremities were thus affected, the rest of the body developed itself as under ordinary circumstances.

The only way that such an occurrence can be explained, is, that the arachnoid, which is a serous membrane, surrounding the spinal cord, by reflex irritation from dentition, became suddenly congested. As a result of this congestion, fluid was effused upon the cord, and its gravitation pressing upon the lower portion, caused paralysis of those nerves distributed to the lower extremities. Now this fluid was more than simply serum, because serum would ultimately have become absorbed. Lymph or fibrin must have been effused, and by becoming organized, maintained its situation and thus kept up the paralysis. In order to relieve the existing trouble, then, supposing this explanation to be correct, the treatment should be only such as would remove the deposit.

We want, therefore, the application of sorbifacient remedies along the spine, such as the veratria ointment. Veratria is a powerful stimulant to the nervous structure, and mercury is likewise a good absorbent, by its action upon the absorbent vessels.

Such ointment, in combination with the hot and cold douche, should be continuously applied, twice daily, for a number of months.

Internally, we can give this child one-quarter of a grain of the iodide of potassium or sodium, in combination with the minute doses of the corrosive chloride of mercury, three times daily, being careful, however, when we use the remedy long, to have intermissions of three, four or five days in the treatment, according to circumstances, so that the stomach may be rested and not rebel against the remedy.

If the system is not well nourished, we can give three drops of the tincture of the chloride of iron, three times daily to a child of this age. It is a powerful tonic, increasing the coloring matter and fibrin of the blood. Huxam's tincture of bark and the syrup of the iodide of iron would also be good remedies in similar cases. The limbs should be thoroughly rubbed; flagellation would also be here indicated.

Notwithstanding what I have said, I am afraid the prognosis here is of the very worst kind.—*Surg. Clinic of Prof. Gross.—Med. & Surg. Reporter.*

